# Effect of Lactic Acid Bacteria Fermentation Agent on the Structure, Physicochemical Properties, and Digestive Characteristics of Corn, Oat, Barley, and Buckwheat Starch

**DOI:** 10.3390/foods14162904

**Published:** 2025-08-21

**Authors:** Ziyi You, Jinpeng Wang, Wendi Teng, Ying Wang, Yuemei Zhang, Jinxuan Cao

**Affiliations:** 1 Key Laboratory of Geriatric Nutrition and Health, Beijing Technology and Business University, Ministry of Education, Beijing 100048, China; 15564060687@163.com (Z.Y.); wenditeng@btbu.edu.cn (W.T.); wang-ying@btbu.edu.cn (Y.W.); zhangyuemei@btbu.edu.cn (Y.Z.); caojinxuan@btbu.edu.cn (J.C.); 2 School of Food and Health, Beijing Technology and Business University, Beijing 100048, China; 3 College of Food and Biological Engineering, Chengdu University, Chengdu 610106, China

**Keywords:** sourdough fermentation, cereal starches, structural properties, functional properties, digestibility

## Abstract

This study modified corn, oat, barley, and buckwheat starches using a Henan-specific sourdough starter, revealing that the initial starch architecture governs differentiated functional transformations. Pore-dominant starches (corn/buckwheat) underwent “inside-out” enzymatic pathways—corn starch exhibited a 38.21% reduced particle size through pore expansion, with long amylopectin chain degradation forming thermally stable gels, establishing it as an ideal base for anti-staling sauces and frozen dough. Buckwheat starch demonstrated a 44% increased amylose content facilitated by porous structures, where post digestion double helix formation elevated the resistant starch (RS) content by 7%, achieving a significant 28.19% GI (Glycemic Index) reduction. Conversely, fissure-dominant starches (oat/barley) experienced “surface-inward” limited erosion—oat starch, constrained by surface cracks, showed amorphous region degradation and short-chain proliferation, accelerating glucose release and adapting it for rapid digestion products like energy bars. Barley starch primarily underwent amorphous zone modification, enhancing the pasting efficiency to provide raw materials for instant meal replacement powders.

## 1. Introduction

Starch, a plant-derived polysaccharide that serves as the primary energy reservoir, undergoes enzymatic hydrolysis in the human digestive system to release glucose for metabolic homeostasis. The glycemic impact of starch is governed by its structural hierarchy: amylose–amylopectin ratios and crystalline organization modulate enzyme accessibility to α-1,4/1,6 glycosidic bonds, thereby controlling hydrolysis kinetics and glucose release [1]. Rising diabetes prevalence has intensified the focus on low-GI (Glycemic Index) foods, where RS plays dual roles—reducing postprandial glycemia through enzymatic resistance and fostering gut health via colonic fermentation into beneficial short-chain fatty acids [2]. The structural engineering of starch granules to limit enzyme–substrate binding sites offers strategic pathways for developing functional foods with optimized digestibility profiles.

Resistant starch modification strategies have been extensively investigated through physicochemical and enzymatic approaches [3]. Among them, both organic acids and complex enzyme modifications can effectively affect the increase in RS with high biosafety. For example, citric acid reacts with starch under humid and hot conditions to form intermolecular diester bonds between substituent groups, thereby improving the resistance of starch to digestion [4]. The treatment of wheat starch by malic acid is able to alter its double helix and crystal structure to promote the production of RS [5]. The use of amylosucrase lengthens the side chains of branched starch and then debranches with pullulanase to generate linear chains. These linear chains are effective at increasing the RS by self-assembling to form nanoparticle-containing microparticles [6].

Sourdough fermentation, as a common fermentation technique in food, mainly consists of mixed flour and water and fermentation agent inoculated for fermentation. It gives fermented dough products a unique sour flavor and aroma while greatly improving their taste, texture, and nutritional value [7]. Sourdough can be categorized into two types based on microbial origin. Traditional sourdough develops through continuous fermentation and colonization by the environmental microbes present in flour and bran, forming stable microbial communities. Due to regional environmental variations, these communities exhibit distinct microbial compositions [8,9]. This type requires the regular replenishment of fermentation substrates and water to maintain microbial activity, resulting in a richer species diversity. Additionally, it demonstrates a high adaptability to harsh fermentation conditions such as a low pH, oxygen limitation, and nutrient depletion. In contrast, starter-inoculated sourdough is fermented with artificially introduced microbial strains, characterized by single-strain dominance but a higher production efficiency. Current research has characterized the microbial composition of sourdough well, predominantly comprising lactic acid bacteria (LAB), with yeasts present in minimal proportions. The LAB community is predominantly heterofermentative, with over 70 identified species including *Lactiplantibacillus plantarum*, *Levilactobacillus brevis*, *Pediococcus pentosaceus*, etc. Sourdough fermentation effectively inhibits starch digestion through dual mechanisms: (1) Microbial amylases (α- and β-amylases) preferentially hydrolyze long chains of amylopectin in amorphous regions, reducing readily digestible components while concurrently releasing short linear fragments that form digestion-resistant double helices; (2) LAB-mediated pH reduction suppresses the activity of digestive amylases, thereby decreasing rapidly digestible starch (RDS) content. *Lactiplantibacillus plantarum* converts the A/B_2_ chains of branched starch into indigestible B_1_/B_3_ chains through chain restructuring dominated by 4 α-glucosyl transferase, significantly improving starch resistance [10]. Lactic acid bacteria fermentation promotes the generation of short linear amylose chains in brown rice, facilitating the formation of double helices that enhance starch digestion resistance [11]. At the same time, fermentation can change the structural properties of starch, which can influence starch physicochemical properties, such as crystallinity, crystalline morphology, and the ratio of straight-chain starch to branched chain starch. This further affects their adhesive and digestibility properties. At the same time, fermentation can give starchy foods better flavor and nutritional properties [12]. For example, *Lactiplantibacillus plantarum* is able to increase the content of starch with short-branched chains and delay starch regrowth [13]. *Bifidobacterium bifidum* is able to improve the thermal properties and regrowth of starch [14].

Current dietary patterns heavily rely on wheat as the primary grain source, leading to nutritional monotony. Diversifying grain utilization—particularly by incorporating underutilized crops—is crucial to enriching consumer choices and improving dietary balance. Corn stands out as a nutritionally valuable alternative. Its starch content serves as a major energy source and key industrial ingredient, while bioactive components like zeaxanthin and phenolic compounds contribute antioxidant properties that support human health. Oats are rich in diverse bioactive components. Specifically, low-molecular-weight glucans in oats competitively occupy enzyme–starch binding sites through non-covalent interactions, thereby reducing amylase activity and suppressing starch digestion [15]. Starch, accounting for approximately 60% of oat’s composition, exhibits unique structural characteristics that influence its functionality: Oat starch consists of distinct A-type and B-type granules, both smaller in size compared with other cereal starches. Its amylose chains are shorter in length and exhibit a higher crystallinity. Branched amylopectin demonstrates a high degree of branching, contributing to its distinctive gelatinization behavior under thermal processing [16]. Barley has attracted growing research interest for its nutritional profile and health-promoting properties. Rich in vitamins, minerals, β-glucans, and essential amino acids often limited in cereal-based diets, it shows potential in mitigating diabetes and cardiovascular risks. Its starch component (47–79% grain weight) demonstrates unique structural–functional relationships; it contains 20–30% amylose, higher than many common cereals. Compared with buckwheat starch, its amylopectin features fewer long branches but abundant short chains. These structural attributes synergistically enhance its water-holding capacity and salt-tolerant gelatinization stability—critical properties for developing functional food and industrial applications [17]. Buckwheat has emerged as a research hotspot due to its exceptional nutritional profile, including a high dietary fiber content and a balanced amino acid composition. Starch, constituting over 70% of its dry weight, serves as the primary nutritional component, with its structural features critically influencing the processing properties of buckwheat-based foods. It has a higher proportion of super-long chains and a dominant A-type crystalline structure [18].

Sourdough fermentation, as a bioprocessing technique driven by complex microbial consortia, has demonstrated significant potential in modulating starch digestibility. However, current research predominantly focuses on wheat-based systems, with a limited exploration of region-specific fermenters applied to underutilized cereals. The Henan sourdough starter—characterized by its unique microbial composition (*Companilactobacillus crustorum*, *Limosilactobacillus ponis*, *Lactiplantibacillus plantarum*, *Levilactobacillus brevis*, *Companilactobacillus farciminis*, etc.)—represents an underexplored regional resource. Critically, how such distinct consortia differentially remodel the structural hierarchy of non-wheat starches remains unknown.

This study bridges this gap by investigating the starch-type-specific effects of Henan sourdough fermentation on four nutritionally distinct cereals: corn, oats, barley, and buckwheat. We hypothesize that the initial structural architecture (e.g., crystalline packing mode, surface porosity) of starch granules governs microbial enzyme accessibility, thereby directing divergent functional outcomes. Our objectives are to (1) decipher the structure–digestibility relationship modified by region-specific fermentation; (2) identify key drivers (e.g., amylose realignment, crystalline degradation) for RS enhancement in each cereal; (3) provide a cereal-tailored modification framework for developing functionally optimized foods.

## 2. Materials and Methods

### 2.1. Materials

Corn, oats, barley, and buckwheat were purchased from Yizhong Seasoning and Vegetable Wholesale Market in Baiyin, Gansu. All chemical reagents (potassium iodide, anhydrous ethanol, etc.) were of analytical grade. The sourdough fermentation starter was obtained from the sourdough making workshop in Henan region. It is made from wheat flour and water through a long process of natural fermentation. The most abundant bacteria in it are *Companilactobacillus crustorm*, *Limosilactobacillus ponis*, *Lactiplantibacillus plantarum*, *Levilactobacillus brevis*, and *Companilactobacillus farcimins* [19].

### 2.2. Preparation of Fermentation Samples

Twenty g of sourdough starter was added to 20 mL of sterile distilled water and activated at 37 °C for 1 h. Subsequently, 100 g each of corn flour, oat flour, barley flour, and buckwheat flour were weighed into sterile beakers. The activated sourdough starter and 100 mL of sterile distilled water were then added to each beaker, adjusting the dough’s DY value to 200. The mixtures were stirred with glass rods until thoroughly mixed, sealed with plastic wrap, and fermented at 37 °C for 24 h, achieving a final lactic acid bacteria colony count of 8 log (CFU/g).

### 2.3. pH and TTA Measurements

Ten g of the fermented sample was collected in a beaker of 90 mL distilled water and stirred magnetically for 30 min. After 10 min of standing, the pH value of the solution was determined using a pH meter. Afterwards, the pH of the solution was titrated with 0.1 M NaOH solution to 8.6 ± 0.05, and the volume of NaOH solution consumed was calculated as the TTA (total titratable acidity).

### 2.4. Separation of Starch

Starch isolation was performed according to the method adapted from Yong et al. [20]. We scrubbed the dough in water, collected the starch slurry, and centrifuged it at 3000× *g* for 20 min. After that, the supernatant was poured off and the yellow-colored precipitated surface was removed. This was mainly pentosan, protein, and collected lower white starch sediment. Distilled water was added and the process was repeated 3–4 times. Then, the precipitate was dried in an oven at 45 °C and ground into powder using a mortar and pestle. Finally, the ground starch samples were passed through a 150 μm sieve and dried in an equilibrium moisture drying dish. This ensured that they reached the same moisture content. The extracted starch was labeled as follows: A1: corn starch unfermented, A2: corn starch fermented, B1: oat starch unfermented, B2: oat starch fermented, C1: barley starch unfermented, C2: barley starch fermented, D1: buckwheat starch unfermented, and D2: buckwheat starch fermented.

### 2.5. SEM

The morphology of the samples was observed using a scanning electron microscope (SU8010, Hitachi High-Tech Corp., Tokyo, Japan). The sample stage was coated with conductive adhesive, and dried starch samples were spread evenly on it afterwards. After applying the gold, the sample images were taken with an accelerating voltage of 3.0 kV at a magnification of ×1000 [21].

### 2.6. Particle Size Measurements

Next, 1 mg of starch was dissolved in 10 mL of water to prepare a 0.1 mg/mL starch suspension. The suspension was then treated with ultrasound at a power of 10 W and a frequency of 10 kHz for 2 min to obtain well dispersed starch particles. The size of starch granules was determined using a laser particle sizer (Mastersizer 3000, Malvern Instrument Co., Ltd., Malvern, UK). The shading after adding the suspension was between 10% and 20% while stirring at 2000 rpm to prevent the starch granules from agglomerating [22].

### 2.7. XRD

The crystalline structure of the starch was determined using an X-ray diffractometer (D8 Advance, Bruker, Madison, WI, USA). Samples were scanned with a step size of 0.033° from 5° to 40° (2θ). The relative crystallinity (RC) of the starch was calculated by evaluating the ratio of the crystalline peak area to the total diffracted area [17], using the software MDI Jade 6.0.

### 2.8. FT-IR

Fourier Transform Infrared Spectroscopy (Nicolet iS5, Thermo Fisher Scientific, Waltham, MA, USA) was used to assess the changes in the short-range ordered structure of the starch. First, 3 mg of starch samples were homogeneously mixed with 300 mg of KBr and then pressed into tablets using a vacuum tablet press. FTIR spectra were acquired using a Thermo Nicolet IS5, and the IR spectrograms were analyzed using OMNIC software (Version 9.0, Thermo Scientific, Madison, WI, USA). The sample powder was scanned 64 times at a wavenumber range from 4000 cm^−1^ to 300 cm^−1^ and a resolution of 4 cm^−1^. The ratio of peak intensities at wavelengths of 1047 cm^−1^ to 1022 cm^−1^ was used to indicate the orderliness of the crystals, and the ratio of peak intensities at wavelengths of 995 cm^−1^ to 1022 cm^−1^ was used to indicate the relative number of double helices.

### 2.9. Thermodynamic Properties

The thermal properties of the starch samples were determined using differential scanning calorimeters (DSC 2500, TA Instruments Instrument Co., Ltd., New Castle, DE, USA). We weighed 3 mg of starch samples and added 9 μL of distilled water. Then, it was sealed in an aluminum crucible using a press and equilibrated with moisture at 4 °C for 24 h. The test was conducted under nitrogen atmosphere and an empty aluminum pot was used as a control for each set of samples. The specific conditions were a temperature increase rate of 10 °C/min and a temperature range of 30 °C to 120 °C.

### 2.10. Pasteurization Properties

The viscosity of the starch samples was analyzed using a rapid viscosity analyzer (RVA-TecMaster, Perten Instrument AB, Hägersten, Sweden), with the method of Zou et al. [23]. Three g of starch sample and 25 mL of distilled water were added to a matching aluminum canister. The program was set as follows: the starch suspension was heated from 50 °C to 95 °C at 12 °C/min, held at 95 °C for 2 min, then reduced to 50 °C at the same rate, and finally held at 50 °C for 1 min, and the test speed was 160 r/min.

### 2.11. Solvency and Solubility

A 0.2 g starch sample (*W*_0_) was suspended in 10 mL of distilled water. The suspension was incubated at 90 °C for 30 min in a water bath. After cooling to 25 °C, the slurry was centrifuged at 3000× *g* for 20 min. The supernatant was transferred to a pre-weighed aluminum dish and dried at 105 °C until a constant weight was obtained (recorded as *W*_1_). The precipitate weight was recorded as *W*_2_. Solubility (%) and water-soluble polysaccharides (WSPs, g/g) were calculated using the following equations:

$$Solubility(S)=\frac{W_{1}}{W_{0}}$$

$$WSP=\frac{W_{2}}{W_{0}(1-\frac{S}{100})}\;(g/g)$$

### 2.12. Proportion of Amylose and Amylopectin Measurements

Take 0.1 g starch, put it in 50 mL volumetric flask, add 1 mol/L potassium hydroxide solution 10 mL, fully dissolve it in 75 °C water bath for 20 min, then cool it and dilute it to the scale. Centrifuge 5 mL of the supernatant plus 25 mL of distilled water. Adjust the pH to 3.0 with 0.1 mol/L hydrochloric acid solution, add 0.5 mL of iodine reagent (100 mL of 2 g KI and 0.2 g I_2_), and then dilute to 50 mL with distilled water. Determine the absorption values of sample solutions λ1, λ2, λ3, and λ4, to obtain ∆A for the sample and a standardized quantitative series comparison. Absorption curves of amylose and amylopectin were plotted using full-band visible scanning with a double-beam spectrophotometer.

### 2.13. Starch Gel Hardness Measurements

The gel texture properties were determined using double compression texture profile analysis (TA.XTC-18, Shanghai Bonson Science & Technology Co., Ltd., Shanghai, China). Samples were collected and poured into Petri dishes (90 mm diameter) in the RVA test. After cooling back to room temperature, the samples were covered and left at room temperature for 12 h to stabilize the gel. Measurement parameters: prediction speed 2.0 mm/s, test speed 2.0 mm/s, post test speed 2.0 mm/s, test distance 10.0 mm, trigger force 5 g, and automatic trigger type.

### 2.14. Starch Digestibility Measurements

Rapidly digestible starch (RDS), slowly digestible starch (SDS), and resistant starch (RS) were determined according to the method of Qin [24]. For this step, 100 mg of starch sample and 10 mL of acetate buffer solution were pasted in a boiling water bath for 20 min. When it was cooled to 37 °C, it was mixed with 10 mL of enzyme suspension containing amyloglucosidase (2500 U/m) and porcine pancreatic α-amylase (3000 U/mL) in a 37 °C water bath. Then, 2 mL sample was collected at 0, 20, 60, 90, 120, 150, and 180 min and mixed with 2 mL of 90% ethanol to inactivate the enzymes. Quantitative glucose release was determined using DNS assay, and the rate of hydrolysis (%) was calculated using the following equations:

$$RDS\;(\%)=\frac{(G_{20}-G_{0})\times 0.9}{S}\times 100$$

$$SDS\;(\%)=\frac{(G_{120}-G_{20})\times 0.9}{S}\times 100$$

RS (%)=(1−RDS−SDS)×100

In the equation, *G*_0_, *G*_20_, and *G*_120_ denote the glucose content of the samples at 0, 20, and 120 min and S denotes the total starch content of the samples. The in vitro digestibility of starch was determined based on the method according to Hamid [25] with minor modifications. Equation (1) was used to calculate the in vitro digestibility of the four cereal starches before and after fermentation.

Ct represents the percentage of hydrolyzed starch at time t (min). C∞ denotes the equilibrium hydrolysis percentage, and k is the hydrolysis rate constant (min^−1^). The hydrolysis index (HI) was calculated as the ratio of the area under the hydrolysis curve (AUC) for fermented grains to that of white bread (reference, HI = 100), as per Equation (1). The Glycemic Index (GI) was then derived from Equation (2).

Ct = C∞ (1 − e^−kt^) (1)

GI = 0.862 × HI + 8.198(2)

### 2.15. Statistical Analysis

Each type of grain flour underwent three separate batches of fermentation. All experiments were conducted in triplicate with results expressed as means ± SD. Statistical significance (*p* < 0.05) was determined using one-way ANOVA with Duncan’s multiple range test in SPSS 19.0 (IBM Corp., Armonk, NY, USA). Data visualization was performed using Origin 2021 (Origin Lab Corp., Northampton, MA, USA).

## 3. Results

### 3.1. pH and TTA

**Figure 1:**
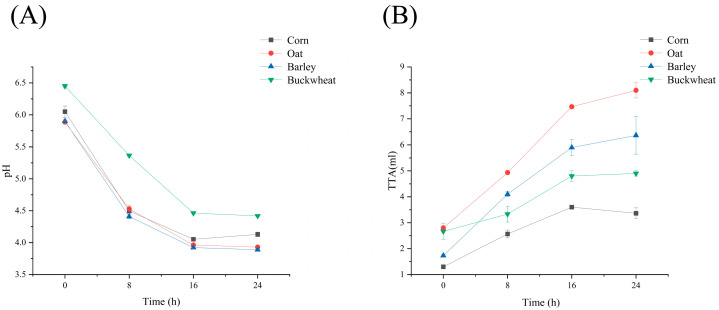
Changes in pH (**A**) and TTA (**B**) with fermentation time.

The pH and TTA (total titratable acidity) changes in corn, oats, barley, and buckwheat after inoculation with the Henan fermentation agent are shown in Figure 1. During fermentation, the pH decreased in all four samples, with the most rapid reduction occurring within the initial 8 h. The pH rapidly decreased to approximately 4.5 due to accelerated microbial metabolic activity. After 24 h of fermentation, the pH stabilized across all four cereals. Oat and barley maintained a pH around 3.9, while corn stabilized at pH 4.1. Buckwheat exhibited the highest pH retention at 4.4. Buckwheat starch shows a higher pH. This may be due to the strong anti-enzymatic action of buckwheat starch. During fermentation, the rate of fermentable sugar release decreases, leading to insufficient substrates for lactic acid metabolism and a higher pH value. This is consistent with the results of higher crystallinity and a higher proportion of RS after buckwheat starch fermentation. At the same time, it has a high content of alkaline amino acids such as histidine and arginine, which give it a high buffering capacity.

As shown in Figure 1B, significant differences in TTA were observed among the four cereals. Oat and barley exhibited pronounced TTA increases, indicating sustained acid production due to efficient substrate utilization and minimal buffering interference. In contrast, corn and buckwheat displayed distinct change patterns: both showed rapid TTA rises during the initial phase (0–16 h), followed by a plateau phase, likely attributable to carbon source depletion or microbial metabolic shifts. Notably, buckwheat demonstrated the narrowest TTA variation range, primarily resulting from its robust buffering system (e.g., the presence of alkaline amino acids).

The pH and TTA were stabilized after 24 h. Corn and buckwheat fermentation yielded lower acid production, whereas oats and barley demonstrated stronger acidification efficacy with higher substrate utilization efficiency, making them suitable for rapidly acidified food production.

### 3.2. Starch Morphology

**Figure 2:**
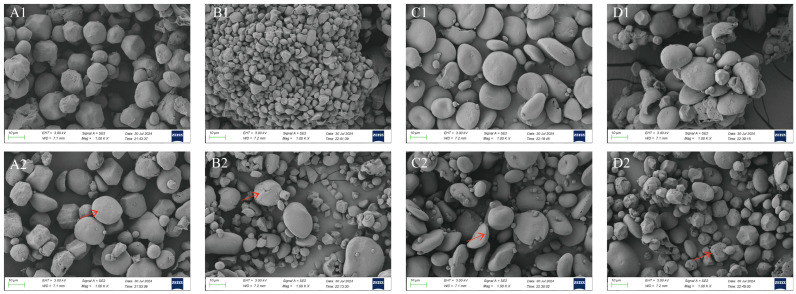
Scanning electron microscopy of starch granules before and after fermentation. (**A1**) corn starch unfermented, (**A2**) corn starch fermented, (**B1**) oat starch unfermented, (**B2**) oat starch fermented, (**C1**) barley starch unfermented, (**C2**) barley starch fermented, (**D1**) buckwheat starch unfermented, (**D2**) buckwheat starch fermented. The position indicated by the red arrow shows the surface damage of the starch after fermentation.

The scanning electron microscope (SEM) images of different starch granules before and after fermentation at a magnification of 1.00 KX are shown in Figure 2. The morphological characteristics of the starch granules varied significantly among the four types. Corn starch presents spherical or irregular polygonal rhombic spheres with rounded edges. The surface of corn starch is smoother and more intact before fermentation [26]. Oat starch has a polygonal or irregular shape. Some of the oat starch granules are formed in clusters and are predominantly irregular, while the others are slightly larger and oval in shape [27]. Most of the barley starch showed a flat, fan, and ellipsoidal shape. Some of the small granules showed a round spherical shape. Buckwheat starch was mostly spherical, cobblestone-shaped, or had an irregular polyhedral morphology with a smooth surface. The four starch granules before fermentation were all relatively intact. Some of them appeared rough, which may be the action of microorganisms carried by themselves during the drying process. Also, the method of separation can have an effect on the structure of starch [28]. Due to the aqueous extraction method of separating starch granules, this resulted in a few non-starch fractions, such as proteoglycans, being attached to the surface of the starch. However, most of the starch granules had smooth surfaces and no significant defects or damage were observed [29].

The corn and buckwheat starch granules after fermentation showed more pronounced surface depressions, holes, and partial fragmentation. This may be due to the action of acid hydrolysis during fermentation and the production of associated amylases for microbial fermentation. Erosion to form porous structures and concave surfaces is the main form of amylase degradation of corn and buckwheat starch granules. At the same time, the fragmentation and collapse of some starch granules occurred. This may be due to the complete contact of porous starch molecules with water molecules. In drying, the dissociation of starch from internal water generates strong forces that break the surface of the granules, resulting in the forced deformation of the granules [30,31]. Structural features such as surface pores and depressions are thought to increase the effective surface area of amylase in contact with the substrate, facilitating enzyme action [32]. After fermentation, slight folds and cracks on the surface of the oat and barley starch granules could be observed. The surface was rough and some of the granules had depressions on the surface. Oat starch before fermentation showed agglomeration, which occurred mainly in small granules and agglomeration decreased after fermentation. This may be due to the fact that oats are rich in gluten and alcohol-soluble proteins. Thereby, forming a gluten network promotes inter-starch aggregation [33]. Proteases secreted by microorganisms during fermentation promote the degradation of the network, thereby reducing aggregation. Barley starch showed more pronounced cracking and fragmentation after fermentation, mainly in the larger starch granules [34].

Collectively, fermentation altered the morphology of the four starches, resulting in structural rupture and surface damage. The corn and buckwheat starches showed more pores after fermentation, which were more effective for enzyme diffusion and adsorption binding. However, the oat and barley starches exhibit cracks. Enzymes or organic acids can only act along the grooves on the surface of starch granules. Thus, the effective area of enzyme action is affected differently. In combination with the different acidity manifestations, the pores have a stronger effect on enzyme binding in fermentation. The corn and buckwheat starches are more sensitive to the action of strains and enzymes in sourdough fermentation agents. In addition, pores and cracks can differentially regulate digestibility. They significantly enhance enzymatic diffusion efficiency during fermentation. Buckwheat starch develops more numerous pores with larger diameters. This causes the more pronounced exposure of internal starch structures, providing additional enzymatic attack sites and yielding the most substantial improvement in digestion resistance—a 21% increase. In contrast, corn starch exhibits smaller pore diameters and shallower depths. Its digestion resistance improved by only 4%. Conversely, cracks dominate the structural changes in the oat and barley starches. Barley displays abundant cracks with partial pore formation along fractures. Oat starch shows minimal cracking and maintains structural integrity, thereby restricting further fermentation-driven modification.

### 3.3. Particle Size

**Figure 3:**
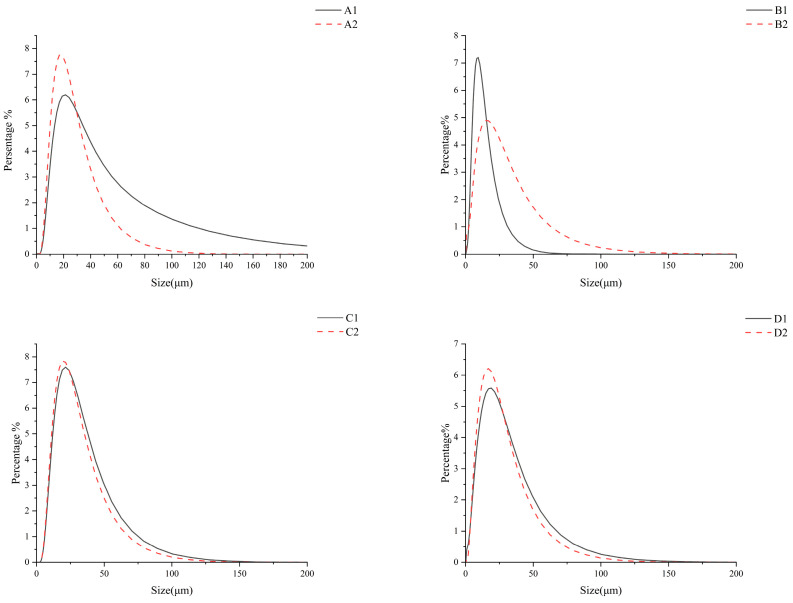
Particle size distribution of starch before and after fermentation. A1: corn starch unfermented, A2: corn starch fermented, B1: oat starch unfermented, B2: oat starch fermented, C1: barley starch unfermented, C2: barley starch fermented, D1: buckwheat starch unfermented, D2: buckwheat starch fermented.

The size of starch granules can respond to the changes in fermentation and also have an impact on the properties and applications of starch. As shown in Table 1, D (4,3) represents the volume-averaged particle size of starch granules. D (0.1), D (0.5), and D (0.9) represent the maximum particle sizes at the 10%, 50%, and 90% thresholds within the starch system, respectively. The D (4,3) of the starch granules varied considerably after fermentation. As shown in Figure 3, compared with the pre-fermentation period, the particle sizes of corn and buckwheat were significantly lower, barley was not significantly lower, and oat was not significantly higher. This may be due to the breakage of starch granules during fermentation. After fermentation, starch is subjected to the action of extracellular enzymes, especially α-amylase, β-amylase, and glycosidase, which hydrolyze the glycosidic bonds on the surface and inside of starch. This leads to the degradation of starch and a reduction in granule size [13].

Enzymatic hydrolysis during fermentation roughened starch granule surfaces and generated fragmented particles, consistent with SEM observations. While reduced granule size typically enhances enzymatic susceptibility through increased surface-to-volume ratios [35], this correlation was absent in fermented systems, indicating size-independent digestion mechanisms. Oat starch exhibited minimal size changes, attributed to limited hydration and protein encapsulation from residual oat components. Concurrently, a high extracellular polysaccharide content promoted granule aggregation, collectively counteracting fragmentation tendencies.

Based on the above, lactic acid fermentation was able to cause a significant reduction in the particle size of the corn and buckwheat starches. After the fermentation of the four cereal starches, oats had the smallest particle size, followed by buckwheat, corn, and barley. Additionally, grain size was not used as a major influence in starch digestion. This phenomenon likely originates from the natural encapsulation of starch by protein networks in cereal grains, which restricts enzyme access to active catalytic sites. For example, the cross-linked mung bean protein significantly reduces the digestion rate of corn starch through dual mechanisms: forming hydrogen bonds and electrostatic interactions with starch molecules, while simultaneously establishing a physical barrier that inhibits starch leaching and blocks amylase accessibility [36]. Concurrently, the aggregation of fine starch particles during fermentation and analytical processing reduces surface area availability, thereby impeding enzyme diffusion into the granular matrix [37]. Consequently, these aggregation effects disrupt the conventional correlation between particle size reduction and enhanced starch digestibility.

### 3.4. Long-Range Ordering XRD

**Figure 4:**
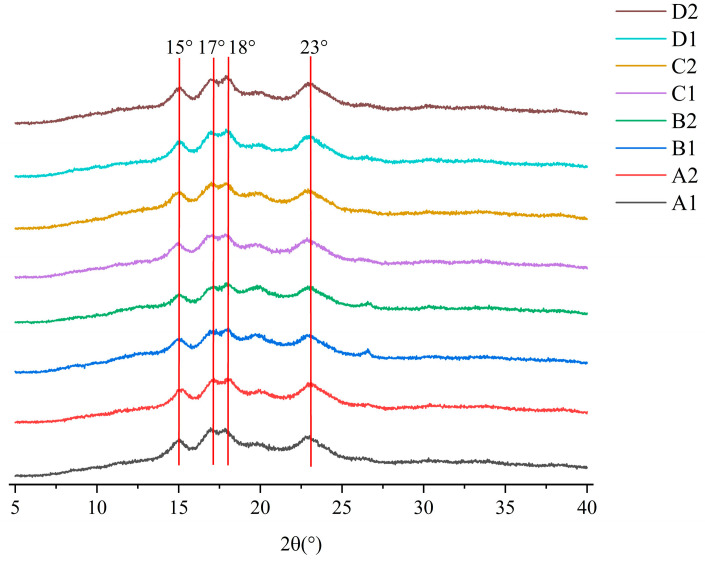
XRD plots of starch before and after fermentation. A1: corn starch unfermented, A2: corn starch fermented, B1: oat starch unfermented, B2: oat starch fermented, C1: barley starch unfermented, C2: barley starch fermented, D1: buckwheat starch unfermented, D2: buckwheat starch fermented.

In Figure 4, XRD analysis revealed preserved A-type crystalline structures in all starches, with characteristic peaks at 15°, 17°, 18°, and 23° remaining post fermentation. The emergence of V-type peaks (13°, 20°) confirmed amylose–lipid complexes. The intensity of the peaks of the four starches varied after fermentation but exhibited the same characteristic peaks. This indicates that fermentation did not change the crystal composition of starch granules. It mainly affects its amorphous region. This mainly results from the fact that the crystalline regions are less susceptible to degradation by the relevant enzymes due to their tightly ordered structural stacking [38].

In Table 2, the RC of starch showed different increases after fermentation. Following fermentation, the RC increased in corn, barley, and buckwheat starches, with corn exhibiting a 5.63% rise. Although oat starch also showed increased crystallinity, the change was minimal at only 0.42%. The increase in crystallinity may be due to amorphous phase breakdown and short-chain realignment [39]. The fermentation-induced cleavage of long-chain amylopectin generates short-chain intermediates, which undergo structural reorganization to enhance crystallinity [40]. This mechanism is amplified by *Lactiplantibacillus plantarum*–*Saccharomyces cerevisiae* co-fermentation, significantly increasing RC [41]. Conversely, oat starch exhibited minimal RC changes due to its inherent structural resistance limiting enzymatic accessibility.

In summary, fermentation did not affect the crystallinity of the starches but increased the RC of corn, barley, and buckwheat starches. This may be due to the degradation of the amorphous regions and the production and rearrangement of short-branched starch.

### 3.5. Short-Range Ordering FT-IR

**Figure 5:**
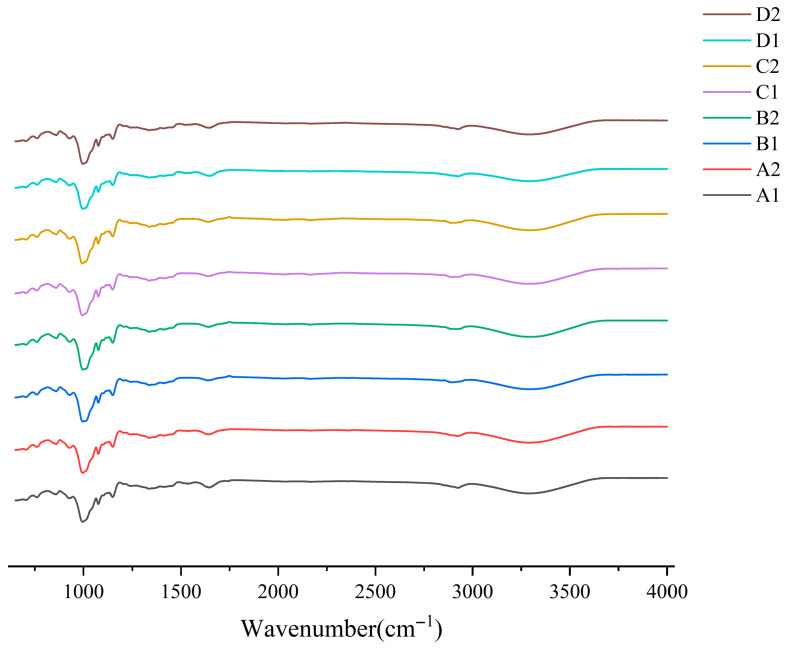
FT-IR before and after starch fermentation. A1: corn starch unfermented, A2: corn starch fermented, B1: oat starch unfermented, B2: oat starch fermented, C1: barley starch unfermented, C2: barley starch fermented, D1: buckwheat starch unfermented, D2: buckwheat starch fermented.

FTIR spectroscopy characterized starch structural features by detecting functional groups and molecular ordering through infrared absorption (400–4000 cm^−1^). All samples exhibited consistent peaks at 3400, 1700, 1370, 1014–1200, and 861–764 cm^−1^ (Figure 5), confirming that fermentation preserved starch’s chemical composition without forming new compounds.

Structural changes were quantified using absorbance ratios: R_1047/1022_ cm^−1^ for molecular ordering and R_995/1022_ cm^−1^ for double helix integrity. As shown in Table 3, the R_1047/1022_ cm^−1^ ratio of starch appeared to decrease after fermentation. The decreasing change in the ratio at R_995/1022_ cm^−1^ was more pronounced in corn and buckwheat starches. This indicates the disruption of the short-range sequence and double helix structure. Combined with SEM, the results indicate that maize and buckwheat starch experience mostly pore erosion. Therefore, the enzyme was able to penetrate deep inside the starch granules until the destruction of the double helix structure formed by the branching of branched starch. At the same time, long-branched starch is degraded to form short-branched chains thus increasing starch disorder. Fermentation-induced starch structural changes exhibit strain- and substrate-dependent patterns. Lactic acid bacteria reduce rice starch crystallinity [42] while LAB–yeast synergy disrupts ordered/double helical structures in buckwheat [43]. Conversely, natural fermentation enhances wheat starch order via hydrogen-bonded microcrystals [44].

In summary, fermentation does not produce new compounds but does increase the disorder of the starch structure. Corn and buckwheat starch fermentation was able to disrupt the double helix structure formed by amylopectin in the crystalline region, adding short-branched chains. Meanwhile for oats and barley, the disruption was mainly in the amorphous region.

### 3.6. Pasting Properties of Starches

The pasting characteristics of starch are a key performance indicator that determines the value of its application in the food industry, and RVA can effectively characterize this process.

**Figure 6:**
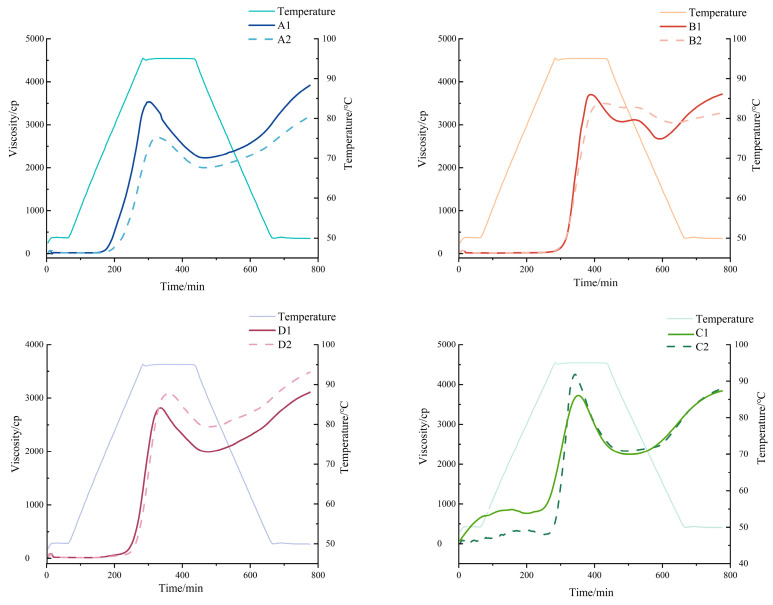
Thermodynamic properties of starch before and after fermentation. A1: corn starch unfermented, A2: corn starch fermented, B1: oat starch unfermented, B2: oat starch fermented, C1: barley starch unfermented, C2: barley starch fermented, D1: buckwheat starch unfermented, D2: buckwheat starch fermented.

In Figure 6, the morphology of the pasting curves of starch was similar before and after fermentation. But, the pasting characteristics (e.g., peak viscosity, disintegration value, and regrowth value) were significantly changed (*p* ≤ 0.05) (Table 4). Corn starch exhibited a 28% decrease in peak viscosity post fermentation (*p* ≤ 0.05), attributable to α-amylase degrading amylopectin into short-chain dextrins that impaired granule hydration capacity. The 50% reduction in breakdown reflected enhanced thermoshear stability. Significant setback reduction stemmed from the steric hindrance of short chains delaying recrystallization. Pasting temperature increased by 26.35 °C due to debranching enzyme-driven amylose enrichment strengthening hydrogen bonding. Final viscosity declined by 18.5%, owing to compromised starch network formation from short-chain dominance. Oat starch showed a 5.5% peak viscosity reduction (*p* ≤ 0.05) primarily through the amylase-mediated cleavage of long amylopectin chains reducing water-binding sites. Breakdown remained stable, indicating preserved structural integrity. Insignificant changes in pasting temperature and setback (<5%) confirmed crystalline region stability during molecular restructuring. Final viscosity decreased 13% due to impaired gel reorganization by short chains. Barley starch demonstrated a 13% peak viscosity increase (*p* ≤ 0.05) through amorphous region disruption, liberating amylopectin for enhanced swelling. Breakdown surged 31%, indicating structural fragility under thermal shear. No significant alterations occurred in pasting temperature, setback, or final viscosity (*p* > 0.05). Buckwheat starch achieved a 8.2% peak viscosity elevation (*p* ≤ 0.05) via amorphous matrix disintegration boosting hydration. Pasting temperature rose 4.2 °C from amylose-driven crystalline reinforcement. Final viscosity increased 10% through strengthened long-chain cold gel networks. Setback and breakdown remained statistically unchanged.

Fermentation restructures starch differently in each grain, creating unique advantages. Corn starch’s short chains block ice crystal growth and slow staling, ideal for extending frozen dough shelf life. Oat starch develops a lower viscosity and a higher GI due to short-chain amylopectin formation, making it ideal for nutritional porridge that delivers quick energy release with a smooth texture. Barley starch thickens better when heated due to structural changes, making it a cost-effective gelatin substitute in hotpot bases. Buckwheat starch gains a higher viscosity with a lower GI, excellent for low-glycemic noodles and thick sauces.

### 3.7. Thermodynamic Properties of Starch

**Figure 7:**
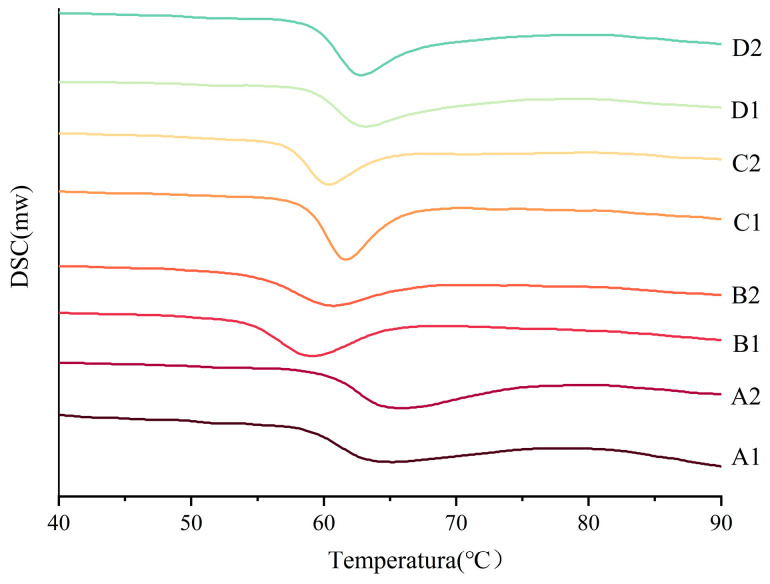
DSC of starch before and after fermentation. A1: corn starch unfermented, A2: corn starch fermented, B1: oat starch unfermented, B2: oat starch fermented, C1: barley starch unfermented, C2: barley starch fermented, D1: buckwheat starch unfermented, D2: buckwheat starch fermented.

DSC characterizes the thermodynamic properties of starch gelatinization, including onset (T_o_), peak (T_p_), and conclusion (T_c_) temperatures, as well as enthalpy (ΔH). As illustrated in Figure 7, cereal starches exhibit a single endothermic transition during heating, corresponding to gelatinization. This process initiates with water absorption and the swelling of amorphous regions, triggering structural disorganization in semicrystalline domains. Subsequent heating disrupts hydrogen bonds in crystalline regions, leading to the uncoiling of amylopectin double helices and the leaching of amylose to form a colloidal solution. Ultimately, the complete structural disintegration of starch granules results in a viscous gel matrix.

As shown in Table 5, the higher the T_o_, the tighter the internal structure of the starch or the greater the resistance to water penetration. After fermentation, corn and oat starch increased, barley starch decreased, and buckwheat starch did not change significantly. The increase in T_o_ of corn and oat starch stems from the fermentation restricting water from entering the interior of the starch granules [45]. This may stem from the residual components of fermentation such as polysaccharides encapsulating the starch, thus limiting the access of water molecules to the interior of the starch granules [46]. Also, the formation of complexes between lipids and straight-chain starch during fermentation can cause an increase in T_o_. For fermented barley starch, water is then more likely to enter the interior. The higher the peak temperature, the more thermal energy is required to destroy the crystalline zone. This may be related to a high amylose content or tight molecular arrangement. The peak temperature of oat starch increased after fermentation and corn and barley starches decreased, whereas buckwheat showed insignificant changes. This suggests that corn and barley starches are more prone to swelling and rupture. The decrease in ΔH values may be due to the degradation of the crystalline regions of the particles, mainly related to the loss of the double helix structure. It suggests that corn, oat, and barley starches have reduced double helix structures after fermentation. The significant decrease in T_c_ − T_o_ after the fermentation of corn starch indicates that the pasteurization of fermented starch occurs more uniformly in a narrower temperature range.

Overall, the effect of fermentation on corn starch was more pronounced. Starch crystallization regions degraded after fermentation. Thermal stability was reduced and more susceptible to fermentation swelling and rupture, but the homogeneity of pasting was increased. The oat starch fermentation process produces enzymes and acids that destroy the amorphous region of the starch, promoting the aging process and making it more tightly recrystallized. Barley starch fermentation breaks down the structural barrier and moisture is more easily accessible to the interior of the granule. This accelerates the destruction of the crystallization zone, and the enthalpy of each temperature drop, and the thermal stability of the starch decreases as a result of fermentation. The enthalpy of buckwheat starch increases after fermentation, and the pasteurization process is delayed.

### 3.8. Starch Swelling Power and Solubility

**Figure 8:**
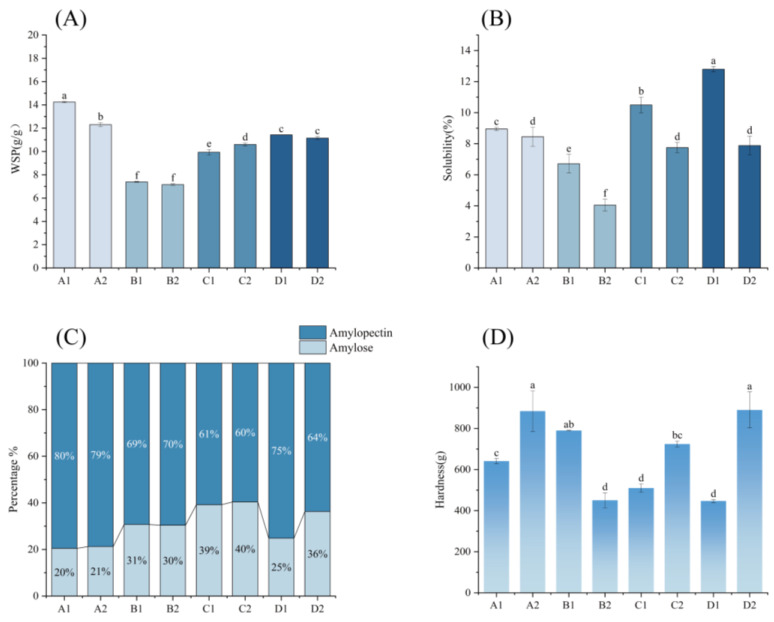
(**A**) The swelling power of starch before and after fermentation. (**B**) The solubility of starch before and after fermentation. (**C**) The ratio of amylose to amylopectin before and after fermentation. (**D**) The gel hardness of starch before and after fermentation. A1: corn starch unfermented, A2: corn starch fermented, B1: oat starch unfermented, B2: oat starch fermented, C1: barley starch unfermented, C2: barley starch fermented, D1: buckwheat starch unfermented, D2: buckwheat starch fermented. ^a–f^ Different letters indicate significant differences between groups (*p* < 0.05).

Starch swelling power and solubility is governed by amylose/amylopectin composition and structural interactions [47,48]. In Figure 8A, corn starch initially exhibited the highest swelling due to its amylopectin-rich structure but declined post fermentation as enzymatic/acidic hydrolysis generated low-molecular-weight sugars that suppressed granule expansion. Oat and buckwheat starches maintained stable swelling, attributed to their amylopectin branching patterns (dominant B_2_/short chains) and amylose entanglement restricting expansion, despite the preserved double helix content. Conversely, barley starch’s swelling increased via edge-specific hydrolysis forming SEM-observed gaps, enhancing water infiltration; therefore, swelling capacity increases [49].

As shown in Figure 8B, all starches exhibited reduced solubility (oat being the lowest). This is mainly due to the hydrolysis of amorphous regions in starch granules. This degradation promoted hydrogen bonding between starch and water molecules, reducing free hydroxyl groups and limiting starch–water interactions during thermal expansion [50]. Additionally, organic acids altered the starch molecular charge states, further suppressing solubility—a trend consistent in both *Lactiplantibacillus plantarum* and natural fermentation [51].

Overall, the solubility of all four cereal starches decreased significantly after fermentation at 90 °C, with oat starch showing the lowest solubility. The changes in swelling power showed different trends due to the differences in starch structure: Corn starch showed a decrease in swelling power due to the increase in surface pores and molecular degradation after fermentation. The swelling power of barley starch increased due to the hydrolysis of the edges to form gaps to promote the penetration of water molecules. Oat and buckwheat starch is rich in straight-chain starch and special branched chain starch structures. Its molecular entanglement and high double helix ratio confer anti-swelling properties, and the swelling force remains stable before and after fermentation. This reveals the relationship between water molecules and starch during the fermentation process and the changes in microscopic morphology.

### 3.9. Proportion of Amylose and Amylopectin

Figure 8C shows the starch composition changes (amylose/amylopectin) in four grains pre/post fermentation. Corn, oat, and barley maintained stable amylose–amylopectin ratios, with corn having the lowest amylose content and barley the highest, consistent with their swelling behaviors. In contrast, buckwheat exhibited a marked amylose increase and amylopectin reduction post fermentation, aligning with its pronounced solubility decline. This shift stems from microbial enzymes and organic acids degrading amylopectin’s short side chains, generating smaller amylose molecules and elevating its proportion [52].

### 3.10. Starch Gel Hardness

As shown in Figure 8D, the hardness of the gel formed by the fermented starch of corn, barley, and buckwheat increased, while the hardness of fermented oat starch gel decreased. Starch gel formation arises from amylose–amylopectin–water interactions: Heating triggers granule hydration and rupture, releasing amylose and amylopectin into a disordered aqueous phase. Upon cooling, long linear amylopectin chains and partial amylose recombine via hydrogen bonds to form a 3D network. Amylopectin stabilizes gel morphology through physical entrapment and hydrogen bonding with water. Gel hardness correlates with amylose/amylopectin content and structure, but primarily depends on amylose proportion—its linearity facilitates ordered alignment, forming a rigid backbone via hydrogen-bonded double helices upon cooling. Fermentation-derived short amylopectin branches further enhance gel strength by filling structural voids [53]. It has been shown that lactic acid bacteria fermentation can increase starch gel hardness [54]. The synergistic fermentation of Saccharomyces cerevisiae and *Lactiplantibacillus plantarum* can effectively increase starch gel hardness [55]. Whereas fermentation reduced the gel hardness of oat starch, this may be due to the fact that fermentation mainly reduced the straight chain amylose content. This is consistent with the results of straight chain amylose content determination.

Overall, fermentation optimized amylopectin structure and enhanced amylose network formation. Corn, barley, and buckwheat starch gels showed significantly increased hardness post fermentation, with corn/buckwheat forming the strongest gels for texture-critical foods (e.g., high-temperature reheating, chewiness). Oat starch’s reduced gel hardness (due to limited amylose release and structural breakdown) suits short-term baked goods requiring soft textures.

### 3.11. Starch Digestibility

**Figure 9:**
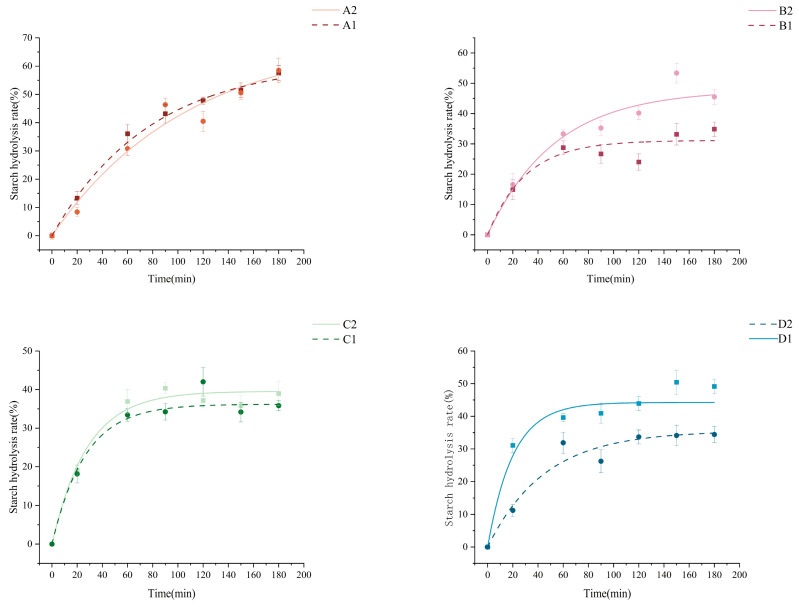
Starch digestibility curves before and after fermentation. A1: corn starch unfermented, A2: corn starch fermented, B1: oat starch unfermented, B2: oat starch fermented, C1: barley starch unfermented, C2: barley starch fermented, D1: buckwheat starch unfermented, D2: buckwheat starch fermented.

As shown in Figure 9, to further evaluate the effect on the starch digestibility and GI value of four grains using the sourdough fermentation agent in Henan Province, China, a first-order nonlinear kinetic equation was used to calculate the kinetic parameters during the digestion process. Initial rapid hydrolysis (<20 min) transitioned to enzyme-resistant phases post fermentation, stabilizing after 120 min, except for corn starch. The sensitivity of starch to contact with enzymes depends on the crystal structure and starch composition. However, during the experimental process, starch was gelatinized to simulate culinary processing, thereby eliminating the influence of native crystalline structures; consequently, fermentation-induced molecular restructuring emerged as the critical determinant of digestion patterns. Post fermentation corn and oat starches exhibited enhanced digestibility, attributable to increased short-chain amylopectin branches that promote enzymatic hydrolysis [56]. These short chains reorganize into tightly packed crystalline domains, elevating relative crystallinity [10], consistent with XRD observations. Conversely, buckwheat’s reduced hydrolysis correlated with an elevated amylose content, which impedes enzymatic access through helical complex formation.

The GI of different grains before and after fermentation are shown in Table 6. The GI of oat starch after fermentation increased significantly from 46.12 before fermentation to 59.96. This may primarily attributed to microbial α-amylase hydrolyzing long-chain amylopectin into more short-chain dextrins, increasing non-reducing ends. These short chains exhibit a stronger affinity for glycosidase active sites, significantly enhancing the enzyme binding efficiency to catalyze rapid glucose generation; concurrently, debranching-released linear amylose fragments undergo rapid conversion to glucose via efficient enzymatic hydrolysis pathways [57]. Buckwheat starch had a significantly lower GI of 49.81 after fermentation than that of 69.37 before fermentation. The GI of corn and barley starches did not change significantly before and after fermentation. The lower GI of buckwheat after fermentation was associated with a higher content of amylose with a lower hydrolysis rate [58]. This is consistent with the results that the proportion of amylose increased after buckwheat starch fermentation.

Based on the changes in Glycemic Index (GI) values, acid dough fermentation enables the directional modulation of cereal starch digestion properties and glycemic response. Fermented buckwheat starch stands as the primary choice for low-glycemic product development—its significantly elevated amylose ratio post fermentation (GI reduced from 69.37 to 49.81) forms enzyme-resistant helical structures that slow digestion, making it ideal for diabetic staple foods and blood sugar-controlling meal replacements. Fermented oat starch emerges as the optimal material for rapid energy release—increased short-chain amylopectin branches (GI elevated from 46.12 to 59.96) and loosened crystalline structures enhance enzymatic efficiency, effectively supporting sports nutrition products and instant energy foods. Corn starch, with an inherent high digestibility and low cost, serves as an economical matrix for energy drinks or meal replacement powders. Barley starch maintains stable GI values before and after fermentation, suiting balanced breakfast cereals. This provides solutions to produce functional foods.

**Figure 10:**
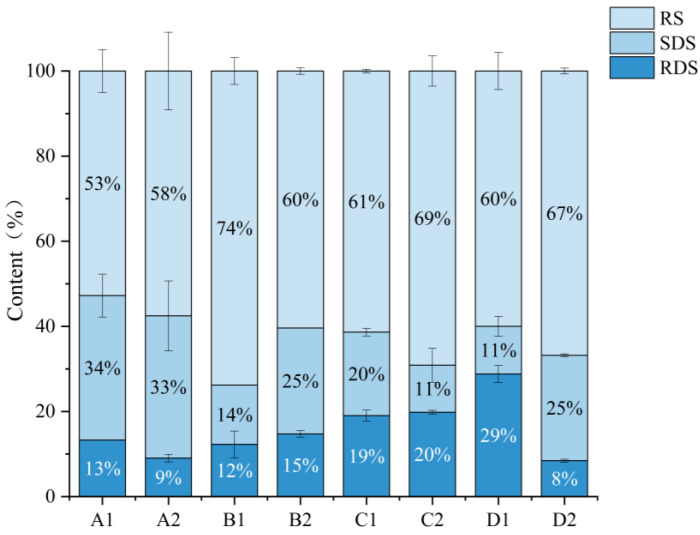
Proportion of starch with different digestibility before and after fermentation. A1: corn starch unfermented, A2: corn starch fermented, B1: oat starch unfermented, B2: oat starch fermented, C1: barley starch unfermented, C2: barley starch fermented, D1: buckwheat starch unfermented, D2: buckwheat starch fermented.

Figure 10 demonstrates the fermentation-modified digestibility profiles of four cereal starches, revealing substrate-specific responses: After fermentation, corn starch had a significantly lower RDS proportion; oat starch had a significantly higher SDS proportion and a significantly lower RS content; barley starch had a significant increase in the proportion of RS and a significant decrease in the proportion of SDS; and buckwheat starch had a decrease in the proportion of RDS and a significant increase in the proportions of SDS and RS. The results showed that there were differences in the sensitivity of the four grains to enzymatic digestion due to fermentation. This was mainly due to the difference in the range of degradation of different cereal starch structures by the fermentation process. The sourdough fermentation agent mainly consists of lactic acid bacteria and yeast [59], and the fermentation process produces various extracellular amylases, such as α-amylase and the debranching enzyme. They promote the hydrolysis of the α-1,6 glycosidic bond of amylopectin and increase the proportion of straight-chain starch [60]. There is a correlation between higher levels of rectilinear starch content and the production of RS. Longer amylose and long-branched chains are cleaved into shorter, essentially linear chains during digestion. These chains may become more resistant to digestion by forming helices to reduce binding sites for enzymes [61]. This is consistent with the increase in straight-chain starch content in buckwheat after fermentation. Meanwhile, the action of some enzymes caused the formation of micropores on the surface of starch granules, which promoted the exposure of the internal structure. When cooled, these pores promote the reorganization of the ordered structure, thus, enhancing the resistance of starch to digestion [62]. Obvious pores can be observed in the SEM images of corn and buckwheat starches after fermentation. Fermentation promoted an increase in the proportion of digestible starch in oats. This suggests that both the amylose in the amorphous region and the long-branched chain starch in the crystalline region [63], which are not easily degraded by digestive enzymes, are destroyed during fermentation. This would increase the digestion rate of oat starch and increase the proportion of digestible starch in oats.

In summary, fermentation can reduce the GI of buckwheat starch and decrease the degree of final hydrolysis effectively. Moreover, it promoted the increased percentages of RS and slow-digesting starch in digestion, mainly due to the increase in the percentage of straight-chain starch. In contrast, fermentation promoted the digestibility of oat starch, increased the proportion of digestible starch, and it was able to disrupt the structure of oat starch to a greater extent and promote the transition to shorter branched chains. Sourdough fermentation agents have greater potential for improving buckwheat starch as a low-GI product. At the same time, the increased RS content and reduced digestibility deliver significant health benefits through dual physiological mechanisms. In glycemic regulation, RS effectively delays enzymatic hydrolysis, converting glucose release from a rapid peak into a gradual gradient pattern—thereby lowering postprandial blood glucose peaks and reducing insulin demand. Concurrently, it enables sustained blood glucose control by modulating hepatic glucose output. For colonic health, undigested RS functions as a slow-release carbon source fermented by microbiota to produce short-chain fatty acids (SCFAs), which have the following functions: acetate enhances gut barrier integrity, propionate suppresses hepatic cholesterol synthesis, and butyrate (with 2–3-fold increased yield) exerts anti-inflammatory effects. This process also promotes probiotic proliferation (e.g., Bifidobacteria), collectively improving metabolic health [64].

### 3.12. Fermentation Effect Analysis

The changes before and after the fermentation of different grains are displayed in Table 7. Corn starch: Fermentation significantly reduced particle size by 38.21% and degraded double helical structures. Pasting temperature surged by 51.51% while breakdown decreased by 52.68%, accompanied by a 37.94% increase in gel hardness and a modest 4.17% reduction in GI. Expanded surface pores facilitated deep microbial enzyme penetration, preferentially hydrolyzing long amylopectin chains and promoting amylose enrichment. This transformed the starch into a high pasting stability cooking base material, where short amylose chains inhibit ice crystal growth and retard retrogradation—ideal for sauces and frozen dough production. Buckwheat starch: Structural disruption (particle size reduced 36.31%) coincided with a 44% amylose increase, 91.06% higher gelatinization enthalpy, 94.68% enhanced gel hardness, 10.22% elevated final viscosity, and 28.19% GI reduction. Deep pore erosion accelerated the enzymatic degradation of starch architecture. The released amylose self-assembles into digestion-resistant double helices during digestion, increasing resistant starch content and establishing buckwheat as an ideal low-GI matrix for diabetic foods. Oat starch: After fermentation, oat starch particle size increased by 26.58%, solubility decreased by 39.71%, disintegration value increased by 11.40%, gel hardness decreased by 42.95%, SDS increased by 11%, and GI value increased by 29.99%. Enzymes only etched surface fragments through cracks, degrading branch points in amorphous regions and weakening internal support structures. Increased short chains reduced inter-chain cross-linking while particle expansion loosened the gel network, accelerating glucose release. This degradation transformed oat starch into a rapid energy release matrix, with a high solubility and low hardness perfectly matching short-baked products like energy bars. Barley starch: The gelatinization enthalpy increased by 100.66%, the peak viscosity increased by 14.38%, the relative crystallinity increased by 11.23% (RC), and the gel hardness increased by 42.05%, but the GI value increased only slightly by 1.02%. Enzymatic action through surface cracks preferentially degraded amorphous components (particularly short branches), accelerating granule swelling and dramatically improving pasting efficiency. This optimization converted barley starch into a high-efficiency instant material ideal for meal replacement powders and instant beverages.

### 3.13. Correlation Analysis

**Figure 11:**
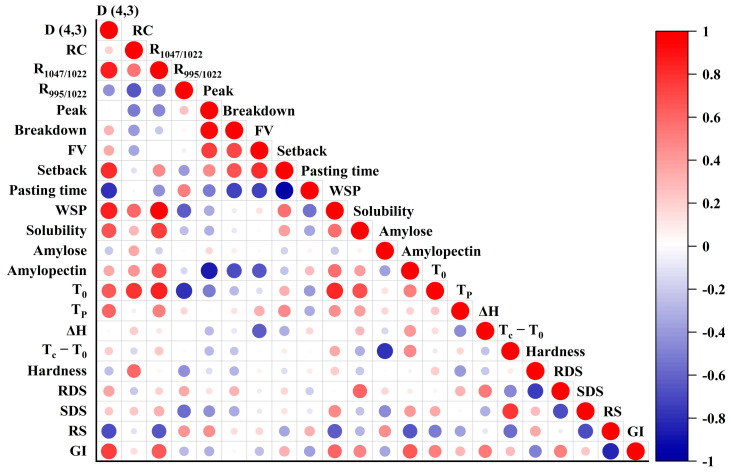
Correlation analysis of all measurements, with red indicating a positive correlation and blue indicating a negative correlation. The intensity of the color reflects the strength of the correlation.

In Figure 11, the results were analyzed in order to assess the correlation between the indicators. It was found that GI was mainly positively correlated with starch disorder, proportion of branched starch, and swelling power and negatively correlated with hardness. RDS was mainly positively correlated with solubility and ΔH. SDS was mainly positively correlated with T_C_-T_O_; RS showed negative correlations with granule size and molecular orderliness, but positive associations with double helix content, peak viscosity, and amylose proportion. Sourdough fermentation primarily degraded amorphous regions, though corn and buckwheat starches exhibited crystalline zone susceptibility due to enzyme-accessible long-chain amylopectin structures. Notably, fermentation effectively lowered GI in buckwheat starch by enhancing RS content. These structural–digestive linkages provide actionable insights for tailoring starch functionality through targeted fermentation protocols, enabling the strategic optimization of glycemic response and digestive profiles in cereal-based products.

## 4. Conclusions

The differential modification of four cereal starches using the Henan sourdough starter reveals the impact of initial structure-directed enzymatic pathways. In pore-dominant starches (corn/buckwheat), fermentation generates enlarged pores, channels, and cavities that facilitate deep microbial enzyme penetration, triggering an “inside-out hydrolysis pathway”. Amylases preferentially degrade long amylopectin chains, releasing abundant amylose fragments. This process effectively reduces solubility and swelling power while enhancing hydrothermal stability. Concurrently, the liberated amylose self-assembles into digestion-resistant double helices during digestion, increasing resistant starch (RS) content and significantly lowering product GI values. Conversely, fissure-dominant starches (oats/barley) exhibit constrained enzyme accessibility due to surface crack structures, resulting in “surface-inward limited erosion”. Enzymatic action focuses on branched amylopectin within amorphous regions, generating increased short-chain fragments. Water molecules infiltrating through fissures accelerate granule disintegration. These short chains are more susceptible to glycosidase degradation than double helices, leading to elevated GI values. These differences are closely related to the initial structure of starch, fermentation-induced enzyme pathways, and colony–substrate interactions, providing a conceptual framework for the targeted development of functional cereal foods.

This study provides insights into how fermented starch structures affect GI values. However, we found some limitations: the sourdough starter contained many microbial species, making it unclear which specific strains caused the effects. Future research will address this limitation through additional experiments to confirm our findings. Further studies should also focus on precise microbial control by isolating key sourdough strains; using gene editing to enhance their ability to create pores or seal cracks in starch for controlled structural changes; developing colon-targeting carriers by loading prebiotics/active ingredients into porous starch; and building machine learning models that predict how starch pores or cracks affect digestion speed to guide low-GI food design. This work will turn regional sourdough resources into standard tools, providing a scientific basis for precision nutrition.

## Figures and Tables

**Table 1:** Particle size distribution of starch before and after fermentation.

Sample	D (0.1)	D (0.5)	D (0.9)	D (4,3)
A1	12.73 ± 1.41 ^f^	26.96 ± 6.87 ^a^	68.71 ± 37.11 ^a^	27.04 ± 5.44 ^a^
A2	19.74 ± 0.38 ^e^	16.70 ± 0.23 ^cd^	36.60 ± 0.81 ^cd^	16.71 ± 0.24 ^bc^
B1	58.92 ± 1.11 ^a^	8.52 ± 0.20 ^e^	21.06 ± 0.27 ^e^	8.05 ± 0.41 ^e^
B2	41.94 ± 2.03 ^b^	12.24 ± 0.56 ^de^	37.01 ± 0.67 ^cd^	10.19 ± 0.70 ^de^
C1	13.08 ± 0.02 ^f^	20.08 ± 0.01 ^bc^	45.06 ± 0.01 ^b^	20.08 ± 0.01 ^b^
C2	14.71 ± 0.18 ^f^	18.77 ± 0.07 ^bc^	40.74 ± 0.15 ^bc^	18.78 ± 0.07 ^b^
D1	25.47 ± 1.07 ^d^	23.40 ± 1.98 ^ab^	39.28 ± 9.50 ^cd^	20.86 ± 0.82 ^b^
D2	33.61 ± 0.59 ^c^	14.01 ± 0.13 ^cde^	35.50 ± 0.12 ^d^	13.29 ± 0.23 ^cd^

A1: corn starch unfermented, A2: corn starch fermented, B1: oat starch unfermented, B2: oat starch fermented, C1: barley starch unfermented, C2: barley starch fermented, D1: buckwheat starch unfermented, D2: buckwheat starch fermented. ^a–f^ Different letters indicate significant differences between groups (*p* < 0.05).

**Table 2:** Relative crystallinity before and after fermentation.

Sample	RC %
A1	35.55 ± 0.91 ^cd^
A2	41.18 ± 1.61 ^ab^
B1	32.57 ± 0.99 ^d^
B2	32.99 ± 0.053 ^d^
C1	34.26 ± 3.07 ^d^
C2	38.11 ± 0.089 ^bc^
D1	37.823 ± 0.33 ^c^
D2	41.72 ± 0.60 ^a^

A1: corn starch unfermented, A2: corn starch fermented, B1: oat starch unfermented, B2: oat starch fermented, C1: barley starch unfermented, C2: barley starch fermented, D1: buckwheat starch unfermented, D2: buckwheat starch fermented. ^a–d^ Different letters indicate significant differences between groups (*p* < 0.05).

**Table 3:** Starch ordering and number of double helices before and after fermentation.

Sample	R_1047/1022_ cm^−1^	R_995/1022_ cm^−1^
A1	0.699167091 ± 0.000457073 ^a^	0.90404789 ± 0.007000453 ^b^
A2	0.688026327 ± 0.00068372 ^b^	0.88342442 ± 0.004103075 ^c^
B1	0.660990101 ± 0.000470347 ^c^	0.92633817 ± 0.002279967 ^a^
B2	0.657804596 ± 0.001815707 ^d^	0.92027157 ± 0.000052704 ^a^
C1	0.678004337 ± 0.00031932 ^e^	0.89634106 ± 0.003683401 ^b^
C2	0.674796404 ± 0.000475477 ^f^	0.90516304 ± 0.00478207 ^b^
D1	0.694524633 ± 0.00040991 ^g^	0.91774859 ± 0.00002986 ^a^
D2	0.682641434 ± 0.00045467 ^h^	0.90101114 ± 0.002253363 ^b^

A1: corn starch unfermented, A2: corn starch fermented, B1: oat starch unfermented, B2: oat starch fermented, C1: barley starch unfermented, C2: barley starch fermented, D1: buckwheat starch unfermented, D2: buckwheat starch fermented. ^a–h^ Different letters indicate significant differences between groups (*p* < 0.05).

**Table 4:** Pasting properties (RVA) of starch before and after fermentation.

Sample	Peak Viscosity	Trough 1	Breakdown	Final Visc	Setback	Peak Time	Pasting Temp
A1	3492.5 ± 143.54 ^c^	2110 ± 33.94 ^cd^	1382.5 ± 109.60 ^bc^	3901.5 ± 99.70 ^a^	1791.5 ± 65.76 ^a^	5.035 ± 0.05 ^f^	51.175 ± 1.38 ^e^
A2	2709.5 ± 19.09 ^e^	2054.5 ± 75.66 ^d^	655 ± 94.75 ^f^	3178 ± 19.80 ^d^	1123.5 ± 95.46 ^c^	5.57 ± 0.14 ^e^	77.525 ± 1.10 ^d^
B1	3706 ± 5.66 ^b^	2649 ± 28.28 ^a^	1057 ± 33.94 ^de^	3699.5 ± 17.68 ^b^	1050.5 ± 10.61 ^cd^	6.47 ± 0.01 ^b^	94.775 ± 0.04 ^a^
B2	3502 ± 25.46 ^c^	2324.5 ± 210.01 ^bc^	1177.5 ± 235.47 ^cd^	3218 ± 84.85 ^d^	893.5 ± 125.16 ^d^	6.935 ± 0.09 ^a^	94.775 ± 0.04 ^a^
C1	3807 ± 42.43 ^b^	2220.5 ± 34.65 ^bcd^	1586.5 ± 77.07 ^b^	3905.5 ± 88.39 ^a^	1685 ± 123.04 ^ab^	5.835 ± 0.05 ^cd^	50.25 ± 0.02 ^e^
C2	4374.5 ± 171.83 ^a^	2297.5 ± 43.13 ^bc^	2077 ± 214.96 ^a^	3806 ± 134.35 ^ab^	1508.5 ± 91.22 ^b^	5.7 ± 0.04 ^de^	51.875 ± 1.66 ^e^
D1	2833.5 ± 20.51 ^e^	2039 ± 63.64 ^d^	794.5 ± 43.13 ^ef^	3149.5 ± 60.10 ^d^	1110.5 ± 3.54 ^c^	5.6 ± 0.01 ^e^	85.6 ± 0.07 ^c^
D2	3067 ± 28.28 ^d^	2415.5 ± 71.42 ^b^	651.5 ± 43.13 ^f^	3471 ± 18.38 ^c^	1055.5 ± 53.03 ^cd^	5.935 ± 0.09 ^c^	88.025 ± 0.11 ^b^

A1: corn starch unfermented, A2: corn starch fermented, B1: oat starch unfermented, B2: oat starch fermented, C1: barley starch unfermented, C2: barley starch fermented, D1: buckwheat starch unfermented, D2: buckwheat starch fermented. ^a–f^ Different letters indicate significant differences between groups (*p* < 0.05).

**Table 5:** Thermodynamic parameters (DSC) of starch before and after fermentation.

Sample	T_o_	T_P_	T_C_	T_c_ − T_0_	∆H
A1	58.535 ± 0.12021 ^c^	65.295 ± 0.03536 ^a^	74.8425 ± 1.34704 ^a^	16.3075 ± 1.22683 ^a^	−8.0275 ± 0.27931 ^de^
A2	60.2383 ± 0.09192 ^a^	57.1 ± 0.32527 ^f^	72.415 ± 0.90745 ^b^	12.1767 ± 0.99938 ^b^	−4.8833 ± 1.09366 ^b^
B1	54.2225 ± 0.07425 ^f^	59.1 ± 0.04243 ^e^	64.9371 ± 0.11373 ^f^	10.7146 ± 0.03948 ^b^	−7.1367 ± 0.17913 ^cde^
B2	54.6733 ± 0.06128 ^e^	60.58 ± 0.11314 ^d^	66.6983 ± 0.24749 ^de^	12.025 ± 0.30877 ^b^	−6.3517 ± 0.0165 ^bcd^
C1	58.6667 ± 0.00471 ^c^	61.835 ± 0.13435 ^c^	65.89 ± 0.12728 ^ef^	7.2233 ± 0.12257 ^d^	−7.57 ± 0.32527 ^de^
C2	57.3217 ± 0.08721 ^d^	60.505 ± 0.17678 ^d^	64.5417 ± 0.29463 ^f^	7.22 ± 0.38184 ^d^	−5.58 ± 0.53269 ^bc^
D1	59.1838 ± 0.20683 ^b^	63.21 ± 0.07071 ^b^	67.6067 ± 0.59868 ^cd^	8.4229 ± 0.80551 ^cd^	−4.3713 ± 0.06894 ^a^
D2	59.3025 ± 0.01061 ^b^	62.94 ± 0.07071 ^b^	68.301 ± 0.39739 ^c^	8.9985 ± 0.38679 ^c^	−8.4033 ± 1.80548 ^e^

A1: corn starch unfermented, A2: corn starch fermented, B1: oat starch unfermented, B2: oat starch fermented, C1: barley starch unfermented, C2: barley starch fermented, D1: buckwheat starch unfermented, D2: buckwheat starch fermented. ^a–f^ Different letters indicate significant differences between groups (*p* < 0.05).

**Table 6:** Starch hydrolysis parameters before and after fermentation.

Sample	C∞ (%)	K × 10^−2^ (min^−1^)	R^2^	AUC	eGI
A1	58.94 ± 2.94 ^b^	1.45 ± 0.12 ^e^	0.95791 ± 0.30 ^a^	68.33 ± 0.80 ^ab^	67.10 ± 0.69 ^ab^
A2	69.45 ± 2.23 ^a^	0.95 ± 0.03 ^e^	0.96314 ± 0.43 ^a^	65.08 ± 0.71 ^b^	64.30 ± 0.60 ^b^
B1	29.99 ± 1.39 ^f^	2.99 ± 0.27 ^c^	0.93754 ± 0.21 ^a^	44.00 ± 2.95 ^d^	46.12 ± 2.54 ^d^
B2	49.19 ± 1.68 ^c^	1.63 ± 0.04 ^de^	0.95433 ± 0.42 ^a^	60.05 ± 2.65 ^c^	59.96 ± 2.28 ^c^
C1	40.1035 ± 1.956 ^de^	3.01 ± 0.49 ^c^	0.98713 ± 0.53 ^a^	58.65 ± 0.68 ^c^	58.75 ± 0.58 ^c^
C2	37.977 ± 0.98 ^e^	4.24 ± 0.49 ^b^	0.98191 ± 0.64 ^a^	59.35 ± 0.48 ^c^	59.35 ± 0.41 ^c^
D1	44.23 ± 2.06 ^d^	5.11 ± 0.13 ^a^	0.9585 ± 0.46 ^a^	70.97 ± 3.09 ^a^	69.37 ± 2.66 ^a^
D2	35.63 ± 1.44 ^e^	2.23 ± 0.36 ^d^	0.95122 ± 0.20 ^a^	48.27 ± 0.42 ^d^	49.81 ± 0.36 ^d^

A1: corn starch unfermented, A2: corn starch fermented, B1: oat starch unfermented, B2: oat starch fermented, C1: barley starch unfermented, C2: barley starch fermented, D1: buckwheat starch unfermented, D2: buckwheat starch fermented. ^a–f^ Different letters indicate significant differences between groups (*p* < 0.05).

**Table 7:** Starch fermentation effect.

	Corn Starch	Oat Starch	Barley Starch	Buckwheat Starch
D(4,3)	−38.21%	+26.58%	−6.47%	−36.31%
R_1047/1022_ cm^−1^	−1.60%	−0.48%	−0.47%	−1.71%
R_995/1022_ cm^−1^	−2.30%	−0.60%	+0.98%	−1.82%
Peak viscosity	−22.48%	−5.53%	+14.38%	+8.23%
Breakdown	−52.68%	+11.40%	+30.87%	−18.00%
Final visc	−19.06%	−12.52%	−2.55%	+10.22%
Pasting temp	+51.51%	0%	+3.23%	+2.83%
RC%	+15.56%	+1.26%	+11.23%	+10.30%
T_o_	+2.91%	+0.83%	−2.29%	+0.20%
T_P_	−12.60%	+2.50%	−2.15%	−0.43%
T_C_	−3.25%	+2.72%	−2.05%	+1.03%
T_c_ − T_0_	−25.26%	+12.24%	+0.04%	+6.83%
DH	−39.15%	+1.12%	+100.66%	91.06%
WSP	−13.63%	−3.13	+6.73%	−2.44%
Solubility	−5.59%	−39.71%	−26.19%	−38.42%
Amylose	+5%	−3.32%	+2.56%	+44%
Amylopectin	−1.25%	+1.45%	−1.64%	−15.33%
Gel hardness	+37.94%	−42.95%	+42.05%	94.68%
RS	+5%	−6%	+8%	+7%
SDS	−1%	+11%	−9%	+14%
RDS	−4%	+3%	+1%	−21%
GI	−4.17%	+29.99%	+1.02%	−28.19%

## Data Availability

The original contributions presented in the study are included in the article; further inquiries can be directed to the corresponding authors.

## Abbreviations

The following abbreviations are used in this manuscript:

LAB	lactic acid bacteria
GI	Glycemic Index
RS	resistant starch
RDS	rapidly digestible starch
SDS	slowly digestible starch
TTA	total titratable acidity
RC	relative crystallinity
A1	unfermented corn starch
A2	corn starch fermented
B1	unfermented oat starch
B2	oat starch fermented
C1	unfermented barley starch
C2	barley starch fermented
D1	unfermented buckwheat starch
D2	buckwheat starch fermented

## References

[1] Li C., Hu Y., Gu F., Gong B. (2021). Causal Relations among Starch Fine Molecular Structure, Lamellar/Crystalline Structure and in Vitro Digestion Kinetics of Native Rice Starch. Food Funct..

[2] Debras C., Chazelas E., Srour B., Julia C., Kesse-Guyot E., Zelek L., Agaësse C., Druesne-Pecollo N., Andreeva V.A., Galan P. (2021). Glycaemic Index, Glycaemic Load and Cancer Risk: Results from the Prospective NutriNet-Santé Cohort. Int. J. Epidemiol..

[3] Zhang Z., Bao J. (2023). Recent Advances in Modification Approaches, Health Benefits, and Food Applications of Resistant Starch. Starch—Stärke.

[4] Arachchi D.M., Halim A., Fadimu G., Farahnaky A., Majzoobi M. (2025). Green Starch Modification Using Citric Acid: Quinoa, Chickpea, and Cassava Starches. Foods.

[5] Mansur A.R., Jeong G.A., Lee C.J. (2022). Preparation, Physicochemical Properties, and in Vivo Digestibility of Thermostable Resistant Starch from Malic Acid-Treated Wheat Starch. Food Res. Int..

[6] Yu J., Zhu B., Dou Y., Wei Y., Tao Z., Zhang L., Zhang T., Yan X. (2025). Formation and in Vitro Digestion of Sorghum Starch-Resveratrol Complex Nanoparticles and the Corresponding Mechanism. Food Chem..

[7] Wang Z., Wang L. (2024). Impact of Sourdough Fermentation on Nutrient Transformations in Cereal-Based Foods: Mechanisms, Practical Applications, and Health Implications. Grain Oil Sci. Technol..

[8] Boscaino F., Ionata E., De Caro S., Sorrentino A. (2024). Non-Conventional Yeasts from Mozzarella Cheese Whey and Artisanal Sourdoughs: Leavening Capacity and Impact on Bread Sensory Profile. Fermentation.

[9] Semumu T., Zhou N., Kebaneilwe L., Loeto D., Ndlovu T. (2024). Exploring the Microbial Diversity of Botswana’s Traditional Sourdoughs. Fermentation.

[10] Xu M., Zou J., Zhao X., Feng Y., Duan R., Yang B. (2022). Effect of Lactobacteria Fermentation on Structure and Physicochemical Properties of Chinese Yam Starch (*Dioscorea opposita* Thunb.). Food Chem..

[11] Yang Y., Liu Y., Duan Z., Tang Y., Shu W., Xie Y., Liu Q., Yuan Y. (2025). Differences in the Chemical Composition and Physicochemical Properties between Brown Rice Kernels and Brown Rice Flours after *Lactobacillus fermentation* and Their Impact on the Qualities of Brown Rice Noodles. LWT.

[12] Cai X., Wijesekara T., Xu B. (2024). New Insights into Recent Development, Health Benefits, Emerging Technologies, and Future Trends of Cereal-Based Fermented Products. Process Biochem..

[13] Chang L., Dang Y., Yang M., Liu Y., Ma J., Liang J., Li R., Zhang R., Du S. (2024). Effects of *Lactobacillus plantarum* Fermentation on the Structure, Physicochemical Properties, and Digestibility of Foxtail Millet Starches. Int. J. Biol. Macromol..

[14] Hong J., Guo W., Chen P., Liu C., Wei J., Zheng X., Saeed Omer S.H. (2022). Effects of Bifidobacteria Fermentation on Physico-Chemical, Thermal and Structural Properties of Wheat Starch. Foods.

[15] Guo D., Xu M., Long D., Shi J., Guo J., Hu Y., Liu S. (2025). Revealing the Influence of Oat β-Glucan on the Structural Properties and Digestive Characteristics of Rice Starch: A Perspective on Different Molecular Weights. Int. J. Biol. Macromol..

[16] Punia S., Sandhu K.S., Dhull S.B., Siroha A.K., Purewal S.S., Kaur M., Kidwai M.K. (2020). Oat Starch: Physico-Chemical, Morphological, Rheological Characteristics and Its Applications—A Review. Int. J. Biol. Macromol..

[17] Obadi M., Qi Y., Xu B. (2021). Highland Barley Starch (Qingke): Structures, Properties, Modifications, and Applications. Int. J. Biol. Macromol..

[18] Basharat Z., Tufail T., Shao F., Virk M.S., Duan Y., Cai M., Hu K., Basharat N., Zhang H. (2025). Sustainable and Contemporary Approaches to Explore the Nutritional and Processing Perspectives of Buckwheat: Current Evidence and Prospects. Food Biosci..

[19] Zheng J., Wittouck S., Salvetti E., Franz C.M.A.P., Harris H.M.B., Mattarelli P., O’Toole P.W., Pot B., Vandamme P., Walter J. (2020). A Taxonomic Note on the Genus *Lactobacillus*: Description of 23 Novel Genera, Emended Description of the Genus *Lactobacillus* Beijerinck 1901, and Union of *Lactobacillaceae* and *Leuconostocaceae*. Int. J. Syst. Evol. Microbiol..

[20] Yang Y., Zheng S., Li Z., Pan Z., Huang Z., Zhao J., Ai Z. (2021). Influence of Three Types of Freezing Methods on Physicochemical Properties and Digestibility of Starch in Frozen Unfermented Dough. Food Hydrocoll..

[21] Davoudi Z., Azizi M.H., Barzegar M. (2022). Porous Corn Starch Obtained from Combined Cold Plasma and Enzymatic Hydrolysis: Microstructure and Physicochemical Properties. Int. J. Biol. Macromol..

[22] Wang N., You Y., Liao X., Zhang F., Kan J., Zheng J. (2023). Ultrasonic Modification of Lotus Starch Based on Multi-Scale Structure: Pasting, Rheological, and Thermal Properties. LWT.

[23] Zou J., Feng Y., Xu M., Yang P., Zhao X., Yang B. (2023). The Structure-Glycemic Index Relationship of Chinese Yam (*Dioscorea opposita* Thunb.) Starch. Food Chem..

[24] Qin Y., Zhang Y., Chen X., Xu F., Zhu K., Wang P., Zhang Y. (2025). Synergistic Effect of Pectin and the Flavanols Mixture on in Vitro Starch Digestion and the Corresponding Mechanism. Food Hydrocoll..

[25] Hamid M.G., Elkhatim K.A.S., Idris Y.M.A., Elsafy M., Rahmatov M., Abdelhalim T., Muneer F. (2025). Impact of Fermentation Time on the in Vitro Enzymatic Digestibility of Traditionally Extracted Pearl Millet (*Pennisetum glaucum*) Starch. LWT.

[26] Wu X., Wu X., Zhang X., Zhang J., Yan X., Zhang Q., Zhang B. (2025). Structural, Physicochemical and in Vitro Digestibility of White Kidney Bean Protein-Corn Starch Complexes under Various Heat Treatments. Food Res. Int..

[27] Feng W., Huang Z., Pan B., Zhang T., Jin Z., Miao M. (2025). Regulation of Naked Oat Starch Structure and Thermal Properties: The Key Role of Phosphorus. Food Biosci..

[28] Zhang R.-Y., Chen P.-X., Liu A.-B., Zhu W.-X., Jiang M.-M., Wang X.-D., Liu H.-M. (2024). Effects of Different Isolation Methods on the Structure and Functional Properties of Starch from Tiger Nut (*Cyperus esculentus* L.) Meal. LWT.

[29] Sun X., Saleh A.S.M., Sun Z., Zhao K., Zhang X., Lu Y., Ge X., Shen H., Li W. (2022). Molecular Structure and Architectural Characteristics of Outer Shells and Inner Blocklets of Normal and Waxy Wheat A- and B- Starch Granules. J. Cereal Sci..

[30] Liu D., Tang W., Xin Y., Yang J., Yuan L., Huang X., Yin J., Nie S., Xie M. (2020). Comparison on Structure and Physicochemical Properties of Starches from Adzuki Bean and Dolichos Bean. Food Hydrocoll..

[31] Zhao Y., Qiao S., Zhu X., Guo J., Peng G., Zhu X., Gu R., Meng Z., Wu Z., Gan H. (2024). Effect of Different Drying Methods on the Structure and Properties of Porous Starch. Heliyon.

[32] Han X., Wen H., Luo Y., Yang J., Xiao W., Ji X., Xie J. (2021). Effects of α-Amylase and Glucoamylase on the Characterization and Function of Maize Porous Starches. Food Hydrocoll..

[33] An D., Li H., Li D., Zhang D., Huang Y., Obadi M., Xu B. (2022). The Relation between Wheat Starch Properties and Noodle Springiness: From the View of Microstructure Quantitative Analysis of Gluten-Based Network. Food Chem..

[34] Lei M., Yuan H., Jia R., Huang Z., Yang Y., Liang Q., Liu X., Pan Z. (2025). Effects of Different Enzymatic Hydrolysis Times on the Structures and Properties of Corn Microporous Starch Particles and Their Applications in Frozen Dough. Food Hydrocoll..

[35] Li M., Daygon V.D., Solah V., Dhital S. (2023). Starch Granule Size: Does It Matter?. Crit. Rev. Food Sci. Nutr..

[36] Wang K., Ge Y., Jia Y., Hou J., Lu F., Liu Y. (2025). Effect of Exogenous Protein Crosslinking on the Physicochemical Properties and in Vitro Digestibility of Corn Starch. Carbohydr. Polym..

[37] Liu G., Zhang R., Huo S., Li J., Wang M., Wang W., Yuan Z., Hu A., Zheng J. (2023). Insights into the Changes of Structure and Digestibility of Microwave and Heat Moisture Treated Quinoa Starch. Int. J. Biol. Macromol..

[38] Xu M., Wang S., Zou J., Qin X., Lv Q., Li B. (2025). Effects of *Lactobacillus plantarum* Fermentation on the Structure and Digestion of Resistant Starch Type 3 and Properties of Fermented Starch in the Simulated Digestion System. Carbohydr. Polym..

[39] Qi Q., Hong Y., Zhang Y., Gu Z., Cheng L., Li Z., Li C. (2020). Combinatorial Effect of Fermentation and Drying on the Relationship between the Structure and Expansion Properties of Tapioca Starch and Potato Starch. Int. J. Biol. Macromol..

[40] Zhang J., Liu Y., Liu M., Zhao Y., Zhu Y., Cui S., Xiao X. (2024). Effects of *Lactiplantibacillus plantarum* Dy-1 Fermentation on Multi-Scale Structure and Physicochemical Properties of Barley Starch. Food Funct..

[41] Liu Y., Danial M., Liu L., Sadiq F.A., Wei X., Zhang G. (2023). Effects of Co-Fermentation of *Lactiplantibacillus Plantarum* and *Saccharomyces Cerevisiae* on Digestive and Quality Properties of Steamed Bread. Foods.

[42] Luo J., Khalid W., Wang L., Li Y., Fan M., Qian H. (2025). Effects of *Lactobacillus plantarum* Fermentation on the Retrogradation Behaviors, Physicochemical Properties and Structure of Rice Starch. Carbohydr. Polym..

[43] Li X., Wei S., Gao Z., Zhao R., Wang Z., Fan Y., Cui L., Wang Y. (2024). The Influence of Cooperative Fermentation on the Structure, Crystallinity, and Rheological Properties of Buckwheat Starch. Curr. Res. Food Sci..

[44] Zhao G., Liu C., Li L., Li J., Wang J., Fan X., Zheng X. (2024). Structural Characteristics and Paste Properties of Wheat Starch in Natural Fermentation during Traditional Chinese Mianpi Processing. Int. J. Biol. Macromol..

[45] Wang Z., Yan J., Ma S., Tian X., Sun B., Huang J., Li L., Wang X., Bao Q. (2021). Effect of Wheat Bran Dietary Fiber on Structural Properties of Wheat Starch after Synergistic Fermentation of *Lactobacillus plantarum* and *Saccharomyces cerevisiae*. Int. J. Biol. Macromol..

[46] Chen S., Qin L., Chen T., Yu Q., Chen Y., Xiao W., Ji X., Xie J. (2022). Modification of Starch by Polysaccharides in Pasting, Rheology, Texture and in Vitro Digestion: A Review. Int. J. Biol. Macromol..

[47] Vamadevan V., Bertoft E. (2020). Observations on the Impact of Amylopectin and Amylose Structure on the Swelling of Starch Granules. Food Hydrocoll..

[48] Jia R., Cui C., Gao L., Qin Y., Ji N., Dai L., Wang Y., Xiong L., Shi R., Sun Q. (2023). A Review of Starch Swelling Behavior: Its Mechanism, Determination Methods, Influencing Factors, and Influence on Food Quality. Carbohydr. Polym..

[49] Wang H., Liu J., Zhang Y., Li S., Liu X., Zhang Y., Zhao X., Shen H., Xie F., Xu K. (2024). Insights into the Hierarchical Structure and Physicochemical Properties of Starch Isolated from Fermented Dough. Int. J. Biol. Macromol..

[50] Zhao Y.-T., Jiang Y.-H., Xin W.-G., Liang M., Song J.-J., Wang C., Chen X.-Y., Suo H.-Y. (2025). Effect of Co-Fermentation with *Lactiplantibacillus plantarum* and *Saccharomyces cerevisiae* on the Structural, Physicochemical, and Digestibility Properties of Lotus Starch. J. Sci. Food Agric..

[51] Bian X., Chen J., Yang Y., Yu D., Ma Z., Ren L., Wu N., Chen F., Liu X., Wang B. (2022). Effects of Fermentation on the Structure and Physical Properties of Glutinous Proso Millet Starch. Food Hydrocoll..

[52] Tang Y., Chen W., Zhu H., Yi C., Yuan J., Liu Y., Li Z., Cheng H. (2023). Digestibility of Indica Rice and Structural Changes of Rice Starch during Fermentation by *Lactobacillus plantarum*. LWT.

[53] Luo H., Dong F., Wang Q., Li Y., Xiong Y. (2021). Construction of Porous Starch-Based Hydrogel via Regulating the Ratio of Amylopectin/Amylose for Enhanced Water-Retention. Molecules.

[54] Ao W., Qin L., Wu N., Ge P., Hu C., Hu J., Peng Y., Zhu Y. (2024). Intensification of Rice Flour Gel Structure by Fermenting Corresponding Rice with *Lactobacillus plantarum*. Curr. Res. Food Sci..

[55] Xie X., Zheng M., Bai Y., Zhang Z., Zhang M., Chen Z., Hu X., Li J. (2023). Effect of *Lactiplantibacillus plantarum* and *Saccharomyces cerevisiae* Fermentation on the Multi-Scale Structure and Physicochemical Properties of Highland Barley Starch. Food Biosci..

[56] Ouyang J., Wang C., Huang Q., Guan Y., Zhu Z., He Y., Jiang G., Xiong Y., Li X. (2024). Correlation between in Vitro Starch Digestibility and Starch Structure/Physicochemical Properties in Rice. Int. J. Biol. Macromol..

[57] Liu X., Xu Z., Liu X., Zhang C., Ma M., Sui Z., Corke H. (2024). Lamellar Structure Changes in Rice Starch during α-Amylase Hydrolysis: Effect of Starch Granule Surface and Channel Proteins. Food Biosci..

[58] Lv X., Hong Y., Zhou Q., Jiang C. (2021). Structural Features and Digestibility of Corn Starch with Different Amylose Content. Front. Nutr..

[59] Lau S.W., Chong A.Q., Chin N.L., Talib R.A., Basha R.K. (2021). Sourdough Microbiome Comparison and Benefits. Microorganisms.

[60] Park J., Park J.-D., Sung J.M. (2025). Effects of Fermentation with *Lactobacillus plantarum* on Rice Flour: The Role of Granular Characteristics. Food Chem..

[61] Liu Q.-Z., Zhang H., Dai H.-Q., Zhao P., Mao Y.-F., Chen K.-X., Chen Z.-X. (2021). Inhibition of Starch Digestion: The Role of Hydrophobic Domain of Both α-Amylase and Substrates. Food Chem..

[62] Liang W., Ding L., Guo K., Liu Y., Wen X., Kirkensgaard J.J.K., Khakimov B., Enemark-Rasmussen K., Hebelstrup K.H., Herburger K. (2023). The Relationship between Starch Structure and Digestibility by Time-Course Digestion of Amylopectin-Only and Amylose-Only Barley Starches. Food Hydrocoll..

[63] Gupta R., Gaur S. (2024). Investigating the Effect of Natural Fermentation in Modifying the Physico-Functional, Structural and Thermal Characteristics of Pearl and Finger Millet Starch. J. Sci. Food Agric..

[64] Jia H., Ren F., Liu H. (2025). Development of Low Glycemic Index Food Products with Wheat Resistant Starch: A Review. Carbohydr. Polym..

